# Genomic footprints of selection in early-and late-flowering pearl millet landraces

**DOI:** 10.3389/fpls.2022.880631

**Published:** 2022-10-12

**Authors:** Adama Faye, Adeline Barnaud, Ndjido Ardo Kane, Philippe Cubry, Cédric Mariac, Concetta Burgarella, Bénédicte Rhoné, Aliou Faye, Katina Floride Olodo, Aby Cisse, Marie Couderc, Anaïs Dequincey, Leïla Zekraouï, Djibo Moussa, Moussa Tidjani, Yves Vigouroux, Cécile Berthouly-Salazar

**Affiliations:** ^1^DIADE, Université de Montpellier, IRD, CIRAD, Montpellier, France; ^2^LNRPV, Institut Sénégalais de Recherches Agricoles (ISRA), Dakar, Senegal; ^3^Laboratoire Mixte International LAPSE, Campus de Bel Air, route des Hydrocarbures, Dakar, Senegal; ^4^CERAAS, Institut Sénégalais de Recherches Agricoles, Thiès, Senegal; ^5^Human Evolution, Department of Organismal Biology, Uppsala University, Uppsala, Sweden; ^6^CIRAD, UMR AGAP Institut, Montpellier, France; ^7^UMR AGAP Institut, Univ Montpellier, CIRAD, INRAE, Institut Agro, Montpellier, France; ^8^DIADE, Institut de Recherche pour le Développement (IRD), Niamey, Niger

**Keywords:** adaptation, flowering, GWAS, genomic scan, yield, *PhyC*, far-red light *FRS12*, *HAC1*

## Abstract

Pearl millet is among the top three-cereal production in one of the most climate vulnerable regions, sub-Saharan Africa. Its Sahelian origin makes it adapted to grow in poor sandy soils under low soil water regimes. Pearl millet is thus considered today as one of the most interesting crops to face the global warming. Flowering time, a trait highly correlated with latitude, is one of the key traits that could be modulated to face future global changes. West African pearl millet landraces, can be grouped into early- (EF) and late-flowering (LF) varieties, each flowering group playing a specific role in the functioning and resilience of Sahelian smallholders. The aim of this study was thus to detect genes linked to flowering but also linked to relevant traits within each flowering group. We thus investigated genomic and phenotypic diversity in 109 pearl millet landrace accessions, i.e., 66 early-flowering and 43 late-flowering, grown in the groundnut basin, the first area of rainfed agriculture in Senegal dominated by dry cereals (millet, maize, and sorghum) and legumes (groundnuts, cowpeas). We were able to confirm the role of *PhyC* gene in pearl millet flowering and identify several other genes that appear to be as much as important, such as *FSR12* and *HAC1*. *HAC1* and two other genes appear to be part of QTLs previously identified and deserve further investigation. At the same time, we were able to highlight a several genes and variants that could contribute to the improvement of pearl millet yield, especially since their impact was demonstrated across flowering cycles.

## Introduction

By the end of this century, the frequency of heat waves in the Sahelian region will increase, with temperatures exceeding the maximums observed over the past century (Battisti and Naylor, 2009; Russo et al., 2016). Global warming has already led to significant crop yield losses in Africa—up to 20% for pearl millet and 15% for sorghum (Sultan et al., 2019). This trend is likely to accelerate with a further 8% yield loss or more predicted in Africa by the 2050s (Roudier et al., 2011; Knox et al., 2012). The main staple food for communities in sub-Saharan Africa, pearl millet (*Cenchrus americanus* (L.) Morrone syn*. Pennisetum glaucum* (L.) R. Br.) is the top-ranked crop in terms of cultivated area in Nigeria (2,000,000 ha), Burkina Faso (1,183,792 ha), Mali (2,164,374 ha), and Senegal (1,023,065 ha) in 2020 (FAOStat, 2016) due to its high tolerance to extreme climate conditions. However, its production remains low, with an average grain yield of 900 kg/ha (Pucher et al., 2015). In the wake of drought periods in the 1970s and 1980s, West African breeding efforts have mostly been focused on developing short-cycle and non-photoperiodic varieties (Matlon, 1990; Niangado, 2001; Evenson and Gollin, 2003). Yet, West African smallholders rely on both early-and late-flowering types (De Rouw and Winkel, 1998; De Rouw, 2004). Despite the drought episodes, late-flowering landraces have been conserved or reintroduced from the 2000s with the trend towards increased rainfall (Lalou et al., 2019). In terms of performance, early-flowering landraces ensure minimum production in the event of a short raining season, while late-flowering landraces benefit from late rains but others specificities such as adaptation to different types of soil, different culinary uses and secondary uses, e.g., fodder, also explaining why farmers conserved both flowering regimes. It has been demonstrated that flowering time highly contributes to the landraces genomic vulnerability to future climatic conditions and thus constitute a major trait for pearl millet adaptation (Rhoné et al., 2020). The pearl millet flowering cycle ranges from very early (<40 days) to very late (>120 days, Haussmann et al., 2006; Upadhyaya et al., 2017). Variations in flowering time are associated with different climatic conditions of cropping and the pattern usually follows a latitudinal gradient (Haussmann et al., 2006; Pucher et al., 2015). While a landrace can be classified as early-or late-flowering, there is still a great deal of heterogeneity. For instance, within landrace full-sib families, differences between the earliest and the latest flowering accessions were reported to be 16 days at minimum and 39 days at maximum; a flowering variability that appears to be genetically controlled (Haussmann et al., 2007, 2012). Landrace variability has already been exploited by farmers to shorten flowering cycles, as a response to the 1970s and 1980s drought episodes, while conserving their varieties (Vigouroux et al., 2011; Dussert et al., 2015). Substantial effort has been made in the past decades to investigate genes associated with flowering cycle in pearl millet (Saïdou et al., 2009; Vigouroux et al., 2011; Lakis et al., 2012; Kumar et al., 2017; Diack et al., 2020). To date, only the role of *PgPHYC* gene has been really validated and some other QTLs were identified (Saïdou et al., 2009; Kumar et al., 2017). Which other genes are involved in the flowering variability of pearl millet landraces remains an open question.

Although the difference in flowering time is one of the primary characteristics between the two types, many differences in other important agronomic traits distinguish the two groups and lead to different uses by farmers as exposed earlier. Late-flowering landraces also have more productive tillers, high-tillering and small panicle, characteristics that can improve drought avoidance strategy (Van Oosterom et al., 2006; Yadav, 2008). Therefore, pearl millet landraces display both individual and populational variability, an asset that needs to be valorized to ensure productivity and stability with the upcoming increase in climate uncertainty in Africa.

We previously used the core collection from Senegal to identify genes involved in the agromorphological differentiation of the two flowering groups (Diack et al., 2020). This study identified a number of genetic markers associated with biomass, but did not identify genes clearly involved in flowering. Here, we adopt a different strategy, by sampling early-and late-flowering accessions that are grown in the same locations, in the groundnut growing area (13–15°N) where they can be cultivated in mixtures allowing gene flow between the two groups (Lalou et al., 2019). By doing so, we minimized the drift effect and maximize the probability of highlighting the regions involved in the differentiation between the two groups. With the objective to identify key genes underlying the phenotypic differentiation between the two flowering groups, we implemented two complementary approaches: (1) genome-wide selection scan to identify key variants showing the highest genetic differentiation between early-and late-flowering landraces, and (2) genome-wide association analysis (GWAS) to detect relevant variants segregating in both groups. This combination of approaches allowed us to identify genes that are strongly involved in flowering including *PgPHYC* and other promising candidates such as *FSR12* and *HAC1*; but also genes related to yield which magnitude of the effects can vary according to the genetic background in which they evolve.

## Materials and methods

### Plant material

At West African scale, three types of varieties according to the cycle length are usually described in pearl millet: early flowering (EF) varieties (70–90 days from sowing to harvest), semi-late flowering (SF) varieties (90–120 days) and late flowering (LF) varieties (120–180 days) (Bezançon et al., 2009). In this study, we sampled individuals from EF varieties, sensitive to photoperiod and adapted to drier zones in Senegal (low rainfall 350–600 mm), and LF varieties, less photoperiod-sensitive type adapted to wetter Sudanian zone (high rainfall 900–1,200 mm) (Niangado, 2001).

**Figure 1:**
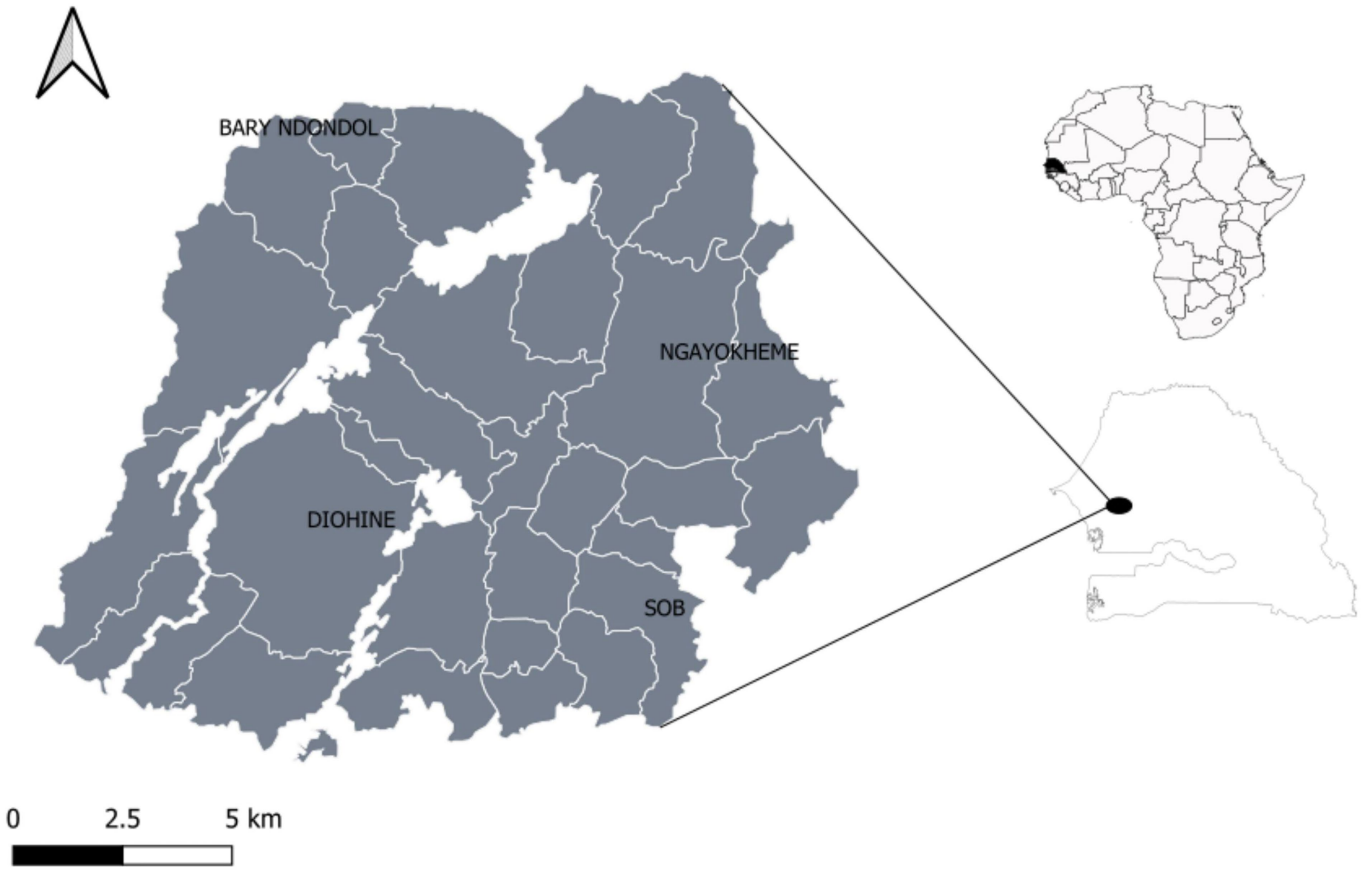
Geographic location of study sites within the Niakhar district.

With the aim of revealing genetic differences between EF and LF pearl millet, we sampled the two flowering groups in Niakhar, an overlapping of the cultivation areas of the two varietal types. This maximizes the probability to detect genes linked to the agro-morphometric characteristics of the two functional groups. Based on information gathered in interviews with farmers, we collected only landraces that had been cultivated in the region for at least 5 years. A total of 109 pearl millet landrace accessions, including 66 early-flowering ‘and 43 late-flowering ‘Sanio’, were collected in four villages in Senegal (Figure 1).

### Phenotyping

Three field experiments were performed in the 2016 rainy season in Senegal and in 2017 in both Niger and Senegal. In Senegal, trials were conducted at the Institut Sénégalais de Recherche Agricole (ISRA) field station in Bambey (14°70′N, −16°47′W). In Niger, the trial was conducted at the International Crops Research Institute for the Semi-Arid Tropics (ICRISAT) field station in Sadoré (13°14′N, 2°17′E). In both areas, the average monthly temperature in June are around 30–35°C during the rainy season, and 20–40°C during the dry season. In the Sahel, millet is generally grown on deep, sandy, red soils (tropical ferruginous soils with little leaching), containing more than 65% sand and less than 18% clay (Swindale, 1982). The trials included three repetitions fully randomized. Eight and 10 individuals per accession for each repetition were sown in Niger and Senegal, respectively. Spacing between each hill was 0.9 m × 0.9 m in the Bambey trial, and 1 m × 0.8 m in the Sadoré trial. To avoid side effects, two rows of cultivated pearl millet were used to border the plots. The sowing dates were 2 August 2016, 21 July 2017 in Bambey and 17 July 2017 in Sadoré. The trials were conducted under rainfall conditions with supplementary sprinkler irrigation when necessary. The Eperon fungicide (3.88% metalaxyl-M + 64% mancozeb) was used at the seedling stage to prevent mildew attacks. Thinning was done to two plants per hill 2 weeks after sowing. All trials were fertilized using the micro-dosing technique (6 g NPK – 15–15–15/hill, corresponding to 93 kg ha^−1^) applied at planting, followed by a 50 kg ha^−1^ urea topdressing after thinning. A total of 9,290 plants were phenotyped for 11 traits associated with plant morphology and fitness: heading date (i.e., number of days from sowing to heading), main stem length, main stem diameter, main panicle length, main panicle diameter, main panicle weight, total seed weight and 1,000 seed weight of the main panicle, total number of tillers and total number of productive and non-productive tillers. For each repetition, the mean trait value was calculated from 6.5 individuals on average after elimination of the minimal and maximal measures.

Broad-sense heritability estimates were calculated as:


$$H^{2}=\sigma_{g}^{2}\times {\left(\sigma_{g}^{2}+\frac{\sigma_{ge}^{2}}{E}+\frac{\sigma_{\varepsilon }^{2}}{ER}\right)}^{-1}$$

with $\sigma_{g}^{2}$ being the genotypic variance; $\sigma_{ge}^{2}$ , the genotype by environment (G X E) variance, and $\sigma_{\varepsilon }^{2}$ , the residual error variance for R replicates and E environments. Analyses were performed using the *mmer* function of the sommer package (Covarrubias-Pazaran, 2016, 2018) in the R environment (Team R Development, 2008). Average values across the nine repetitions for 11 traits of 109 accessions were used to perform a centered and scaled principal component analysis (PCA) and to test for phenotypic differences between EF and LF landraces using the R software ade4 package (Dray and Dufour, 2007).

### Genotyping

#### Exome capture

An exome capture approach was used to target gene-bearing regions of the pearl millet genome. NGS libraries were prepared as previously described (Mariac et al., 2014). Enrichment by capture was performed as recommended by the provider using myBaits kits (Arbor Biosciences) but a single dose of bait was used to enrich the bulk of 48 normalized libraries. Probes were 80 bp long with 80 bp spacing (0.5 x tilling). Probes were designed on the 37,617 mRNA sequences of the pearl millet genome (version v1.0) using the annotation file (pmassemblyv1.020140816.gff available at http://dx.doi.org/10.5524/100192). Contig and scaffold sequences were excluded. For each mRNA target, only the first 1,000 bp of mRNA (starting from the 5′ of the annotation) was used to design up to seven capture probes. A final set of 152,619 probes from 31,895 loci was obtained. The total baited length was 12,209,520 bp, which covered 13.4% of the initial target loci. Enriched libraries were paired-end sequenced (2 × 150 bp) on an Illumina Hiseq2000 platform at Genotoul, Toulouse, France.

#### SNP calling, SNP filtering, and annotation

Demultiplexing based on 6 bp barcodes was performed using the freely available PYTHON script DEMULADAPT (https://github.com/Maillol/demultadapt), using a zero mismatch threshold. Adapters were then removed with Cutadapt 1.2.1 software (Martin, 2011) using a quality cut-off of 20, a minimum overlap of 7 and a minimum remaining length sequence of 35 bp. Reads with a mean quality of under 30 were discarded thereafter using a freely available PERL script (https://github.com/SouthGreenPlatform/arcad-hts/blob/master/scripts/arcad_hts_2_Filter_Fastq_On_Mean_Quality.pl). Reads were mapped to the pearl millet genome (Varshney et al., 2017) with BWA mem v0.7.2 (Li and Durbin, 2009). Only properly paired reads were kept. We used the rmdup module from Samtools 0.1.17 (Li et al., 2009) to remove duplicate reads. RealignerTargetCreator and IndelRealigner from GATK 2.4.7 (DePristo et al., 2011) were used to realign indels. SNPs and genotypes were called using UnifiedGenotyper and the resulting VCF file was filtered for biallelic SNPs only, clustered SNPs (no more than three SNPs per 10-bp window), and mapping quality (MQ0 ≥ 4 && ((MQ0/(1.0*DP)) > 0.1). Obtained raw SNPs were filtered out for low coverage (−min-meanDP ≤4) or excessively high coverage (−min-meanDP ≥100); for low quality (QUAL <60); for quality by depth (QD <2.0); for mean quality (MQ ≤ 40.0); and for Fisher strand score (FS ≥ 60). Finally, SNPs with less than 50% missing data were kept. The final VCF file contained 196,581 SNPs with an average depth per site per individual of 11.057. The maximum individual missing rate was 12.4%. To annotate SNPs and predict their effects, the genome annotation files available at http://dx.doi.org/10.5524/100192 (Varshney et al., 2017) and SNPeff 4.3 (Cingolani et al., 2012) were used considering a maximal distance of 1,000 bp for the SNP to be associated with a gene (Supplementary Tables 1, 2). Based on the available annotation (Varshney et al., 2017), we retrieved, when possible, homologous genes in *Arabidopsis thaliana* and their annotation that were extracted from the TAIR database (https://www.arabidopsis.org/, Supplementary Table 6).

### Genomic diversity and structure

Genetic diversity was estimated by calculating the observed heterozygosity (*H*_OBS_), expected heterozygosity (*H*_EXP_), inbreeding coefficient (*F*_IS_), and differentiation (*F*_ST_; Weir, 1996) between flowering groups using a homemade script.

To evaluate the genetic structure of our dataset, we first ran a PCA using the R package ‘SNPRelate’ v.1.16.0 (Zheng et al., 2012). We further examined genomic clustering patterns using the sNMF function (Frichot et al., 2014) implemented from the R package ‘LEA’ v.3.1 (Frichot and François, 2015). The number of *K* clusters was allowed to vary between 1 and 10. The estimation procedure for each assumed *K* was replicated tenfold. The *K* value and its best run were retained based on the lowest cross-entropy value (Frichot and François, 2015).

### Selection and phenotypic association

#### Genomic scans for outlier SNP detection

In order to detect outlier SNPs, two different genomic scans implemented in the R package ‘PCAdapt’ v.4.1.0 (Luu et al., 2017) and in BayeScan v.2.1 software (Foll and Gaggiotti, 2008) were performed. Both approaches are geared towards identifying SNPs with extreme genetic differentiation values between gene pools. PCAdapt performs a PCA and calculates, for each SNP, a statistic that measures the proportion of genetic variance explained by the first *K* principal components. SNP showing significantly higher loads on the *K* axis are pinpointed as outliers. BayeScan quantifies the biological processes (migration rates, drift) of a demographic history that would lead to the observed allelic frequency variation between populations. BayeScan analysis involves first defining *a priori* populations in order to estimate the allele frequencies of the ancestral population. Outlier SNPs are identified as SNPs that show between-population differentiation that cannot be explained by the modeled demographic history. Each method has its own assumptions and hypotheses and thus is not sensitive to the same biological features. Comparing the results of the two detection methods and keeping only SNPs detected by both can be an efficient way to limit false detections.

We applied the PCAdapt method to SNPs with a MAF higher than 5% (*n* = 96,801) and retained the first principal component (*K* = 1), which differentiated EF and LF accessions. We used the Mahalanobis distance test statistics and applied a false discovery rate (FDR) of 0.001 to identify candidate outlier SNPs. All methods used (PCA on phenotypic or genetic data; sNMF) assigned accessions to the same and expected flowering group. This EF and LF classification was thus used to apply the BayeScan method. We set the prior odds of the model with selection at 10,000, with a thinning interval of 20 and a FDR of 0.05. High prior odds were applied to reduce the false-positive rate but at the expense of missing true loci under selection (Foll and Gaggiotti, 2008).

#### Genome-wide association study

To gain further insight into the genetic variants involved in relevant phenotypes and their variability within each flowering, a genotype–phenotype association analysis of each SNP with the 11 agronomic traits was performed. Three different models for this analysis were used: (i) an efficient mixed-model association (EMMA) implemented in the R package ‘emma’ (Zhou and Stephens, 2014); (ii) a latent factor mixed model (LFMM) implemented in the R package ‘lfmm’ v2 (Caye and Francois, 2018); and (iii) the compressed MLM (CMLM) model implemented in the R package GAPIT v2016.03.01. Kang et al. (2008) developed an efficient mixed-model association (EMMA) method that includes an identity-by-state allele sharing kinship matrix to control for neutral genetic background. Latent factor methods such as LFMM (Frichot et al., 2013), the generic relationship matrix can be described by *K* latent factors (analogous to the principal components in PCA) and can account for more subtle population structures (de Villemereuil et al., 2014; Lotterhos and Whitlock, 2015). CMLM model was run by taking in consideration the kinship and the population structure matrices. For structure, a PCA with two principal components was used. SNPs with MAF below 0.05 were filtered and missing values were imputed using the available function in the LEA v3.1 package (Frichot and François, 2015; Gain and François, 2021). The three models (LFMM, EMMA, CMLM) were implemented for each of the nine repetitions (3 trials × 3 repetitions). The resulting *p*-values of each trial were combined using a Fisher’s combined probability test and associations were considered significant if the Fisher’s combined p-values were < 10^−8^. For each SNP, the allelic effect on the heading date was estimated. A mixed linear model (MLM) using the GAPIT R package (Lipka et al., 2012) including, as co-factors, either the kinship matrix obtained from EMMA or a matrix derived from the latent factor matrix estimated by LFMM was used. The following transformation was applied to derived a kinship-like matrix from the latent factors matrix:


$$K=\frac{UU^{T}}{n}$$

where, *U* is the matrix of latent factors and *n* is the number of genotypes. We then extracted the effect estimated by the mixed model. For CMLM, the effects were directly extracted from the GAPIT implementation of the model, using the same kinship matrix and assuming two principal components of a PCA to describe genetic structure.

## Results

### Phenotypic diversity and clustering

**Figure 2:**
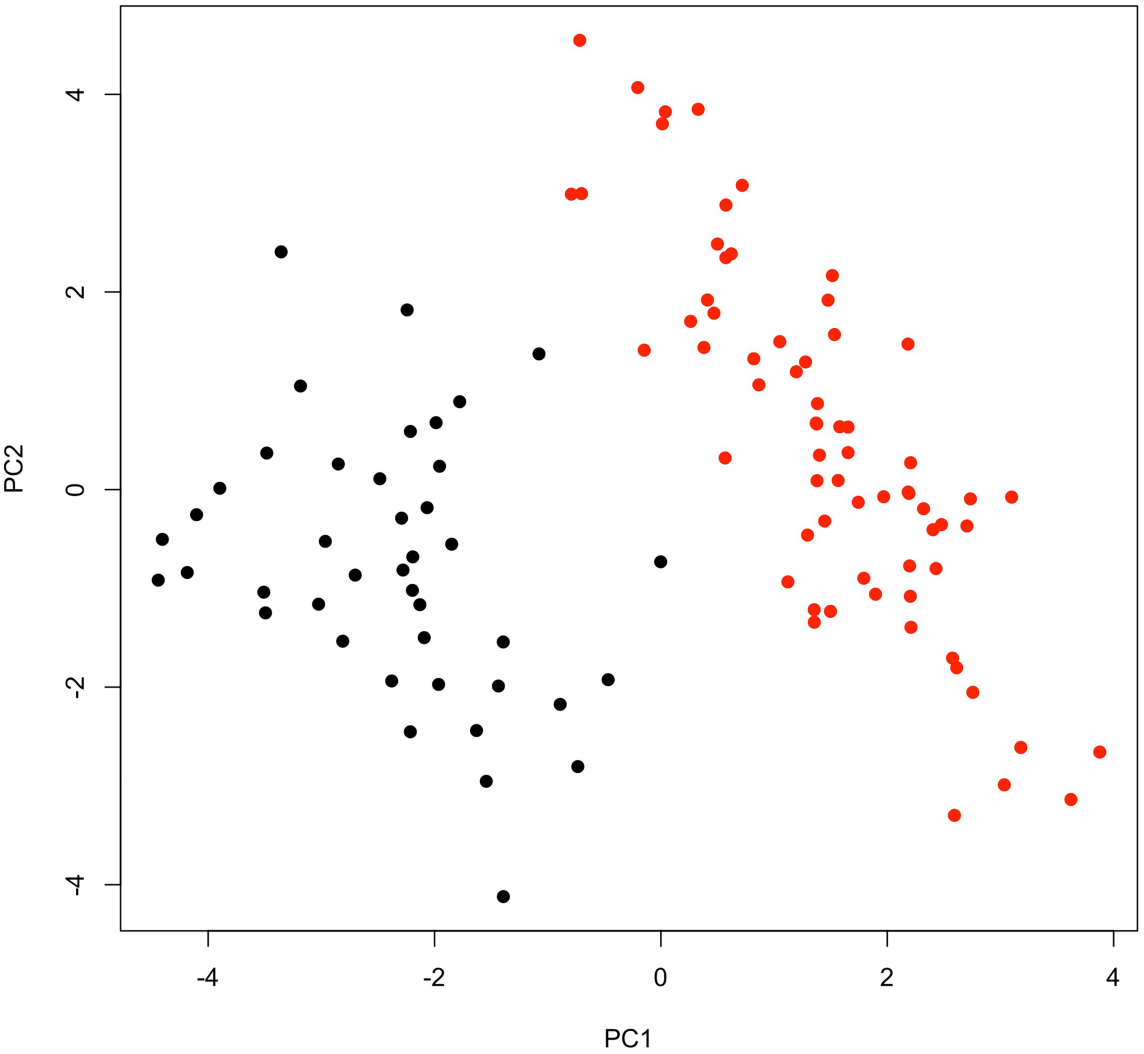
Phenotypic differentiation for early- and late-flowering pearl millet accessions. Mean phenotypic measures across nine repetitions for 11 traits of 109 accessions were used to perform PCA. PC1, and PC2 explained 41.5 and 28% of the inertia, respectively. Early- (red) and late-flowering (black) accessions are well separate on the first PCA axis.

Broad sens heritability for heading date was very high (0.99), as well as for panicle length (0.94). The lowest heritability values were found for the number of non-productive tillers (0.53) and seed weight (0.55, Table 1). All traits were significantly different between early-flowering (EF) and late-flowering (LF) accessions (Table 1; Wilcoxon test, *p* < 0.05). All but 10 pairwise correlations between traits were significant (Supplementary Table 3). Heading was positively correlated with stem size (*r* > 0.77) and number of tillers (*r* > 0.6). For EF accessions, heading occurred on average 52 ± 2.1 days after sowing, which was 30 days earlier than for LF accessions (82 ± 2.6; Table 1). This marked differentiation in cycle length measured in terms of average number of days to 50% flowering after sowing is observed in most studies (Ouendeba et al., 1995; Haussmann et al., 2007; Pucher et al., 2015; Sy et al., 2015). These two maturity categories are found for most varieties in West Africa, in other regions early maturity is less than 50 days from flowering (Lakis et al., 2011). EF accessions showed shorter stems (240.2 ± 12.2), fewer tillers (4.1 ± 0.6) but longer panicles (61.7 ± 7.5) than LF accessions with longer stems (277.8 ± 17.7), more tillers (5.3 ± 0.7) and shorter panicles (54.2 ± 4.5). These results are similar to those of Akanvou et al. (2012) and Dancette (1983) who indicate that LFs are taller. This small size of EFs could be responsible for their greater drought tolerance marked by significant differences in stomatal conductance mainly in leaves (Ghatak et al., 2016, 2021). In addition to these quantitative characters, the LFs spikes aristation is a qualitative character allowing to distinguish LFs to EFs. The principal component analysis (PCA; Figure 2; Supplementary Figure 1) also revealed a clear morphological distinction between EF and LF accessions as largely captured by PC1, which explained 41.5% of the inertia. As expected, the heading date highly contributed to the phenotypic differentiation (PC1: 15% and PC2: 6%; Table 1), but other traits also seemed important for differentiation between the two groups, such as the number of productive tillers (PC1: 17%), panicle weight (PC1: 7% and PC2: 18%) and panicle diameter (PC2: 23%).

**Table 1:** Phenotypic diversity Heritability estimates (±SD) and mean values of phenotype diversity (±SD) in early- (EF) and late-flowering (LF) pearl millet accessions are given.

Trait name	*H* ^2^	EF	LF	PC1 (%)	PC2 (%)
Heading date (days)	0.99 ± 0.001	52 ± 2.1	82.6 ± 2.6	15	6
Stem length (cm)	0.74 ± 0.04	240.2 ± 12.2	277.8 ± 17.7	8	9
Stem diameter (cm)	0.84 ± 0.03	1.6 ± 0.1	1.8 ± 0.1	4	21
Panicle length (cm)	0.94 ± 0.01	61.7 ± 7.5	54.2 ± 4.5	9	2
Panicle diameter (cm)	0.84 ± 0.03	2.4 ± 0.2	2.5 ± 0.1	0	23
Panicle weight (g)	0.66 ± 0.04	70.8 ± 9.7	65.7 ± 6	7	18
1,000 seed weight (g)	0.73 ± 0.04	7.5 ± 0.5	6.7 ± 0.5	13	1
Seed weight (g)	0.55 ± 0.07	41.4 ± 6.1	37.8 ± 4	8	15
Tiller number	0.62 ± 0.07	10 ± 0.8	12 ± 1.2	15	1
Number of productive tillers	0.73 ± 0.05	4.1 ± 0.6	5.3 ± 0.7	17	0
Number of non-productive tillers	0.53 ± 0.07	5.7 ± 0.6	6.1 ± 0.8	3	4

We reported the proportion of variable contributions to PC1 and PC2. 17% of the differentiation explained by PC1 was due to the number of productive tillers. 23% of the differentiation explained by PC2 is due to the panicle diameter trait.

### Genomic diversity and structure

After applying different VCF filters, a total of 196,581 variants were identified with an average depth per site per individual of 11.057. All variants were within or close to 20,126 genes (Supplementary Table 2). Among these SNPs, 96,881 SNPs had a minimum allele frequency higher than 5% (MAF > 0.05, Supplementary Table 4). The average genomic expected and observed heterozygosities seemed slightly higher in EF accessions (*H*_EXP_: 0.167; *H*_OBS_: 0.167) than in LF accessions (*H*_EXP_: 0.155; *H*_OBS_: 0.158). The mean genetic differentiation *F*_ST_ was low but significant at 0.048.

**Figure 3:**
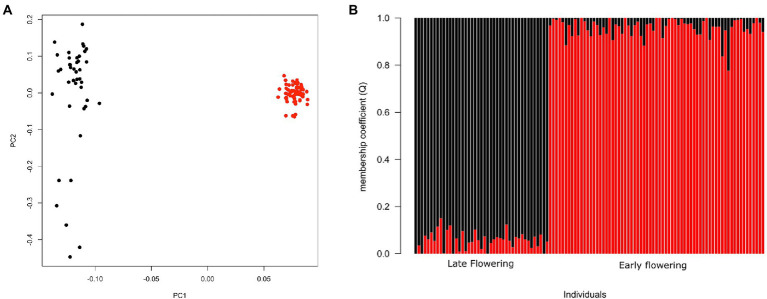
Genomic differentiation for early and late-flowering pearl millet accessions. **(A)** Principal component analysis (PCA) of pearl millet showing first and second principal components. **(B)** Genetic structure for *K* = 2 using sNMF. Each individual is represented by a vertical bar, partitioned into *K* segments representing the amount of assignment of its genome in *K* clusters identified by different colors. Red circles and bars correspond to EF accessions and black circles and bars characterize LF accessions.

The first two principal components of the PCA obtained from the genomic dataset explained 5.7 and 1.3% of the inertia, respectively (Figure 3A). The first axis (PC1) separated EF from LF accessions while PC2 explained the diversity among LF accessions. The lowest cross entropy value for sNMF clustering analysis was obtained for *K* = 2 (Figure 3B), separating EF from LF accessions. All but one individual had a high membership coefficient (≥0.80) with respect to their corresponding flowering group. Overall, PCA and sNMF results were largely congruent, indicating that the phenotypic groups of EF and LF landraces corresponded to highly distinguishable genetic groups.

### Genomic scans for SNP outliers

**Figure 4:**
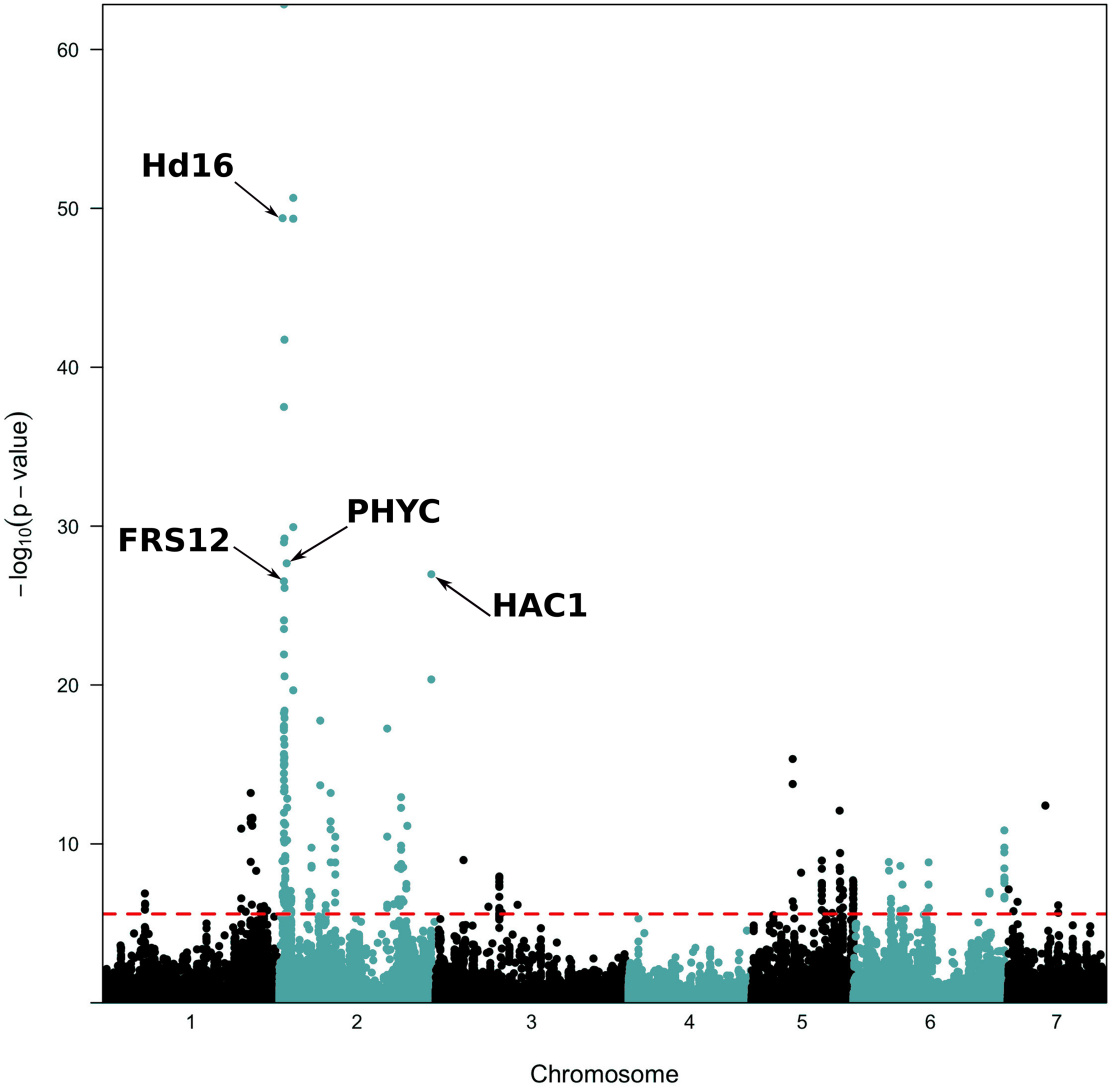
Manhattan plots of *p*-values for PCAdapt analysis along the genome. The analysis was performed on the 96,881 SNPs with a MAF ≥ 5%. The red dashed line represents the 0.001 false discovery rate (FDR) value considered for significance.

The PCAdapt analysis highlighted 253 outlier SNPs, with 54% of them localized on chromosome 2 and 19% on chromosome 5 (Figure 4). BayeScan analysis identified 23 outlier SNPs, while they were all also detected in the PCAdapt analysis (Supplementary Figure 2). All common outlier SNPs were nearly fixed in one variety and absent in the other one (*F*_ST_ > 0.88; Table 2). A total of 21 of these 23 SNPs localized in 12 genes on chromosome 2. The two remaining SNPs were in one gene that was found on chromosome 5. 12 of the 21 SNPs on chromosome 2 co-localized in a single 45 kb window (from 6,908,254 to 6,951,748). Among the 13 genes detected by both selection methods, four were linked to flowering time variation in pearl millet or in other species: *PHYTOCHROME C* (*PhyC*, *Pgl_GLEAN_10016106*); *HEADING DATE 16/EARLY FLOWERING 1* (*Hd16/EF1*, *Pgl_GLEAN_10033790*); *FAR1-RELATED SEQUENCE 12* (*FRS12*, *Pgl_GLEAN_10004675*) and a *HISTONE ACETYL TRANSFERASE 1/HAC703* (*HAC1/HAC703, Pgl_GLEAN_10020525*).

**Table 2:** List of the 23 SNPs detected by both PCAdapt and BayeScan methods. Reference and alternate alleles are provided.

SNP_name	Ref/Alt	*F* (EF)	*F* (LF)	*F*_ST_	Gene	Annotation
chr2_167825475	C/A	0.92	0.03	0.888	Pgl_GLEAN_10003827	NA
chr2_6951644	G/T	0.99	0.03	0.958	Pgl_GLEAN_10004671	NA
chr2_6951583	A/C	0.99	0.06	0.936		
chr2_6951364	A/G	0.99	0.09	0.901		
chr2_6950866	A/G	0.97	0.05	0.915		
chr2_6951748	C/A	0.98	0.08	0.896		
chr2_6940299	T/C	0.97	0.05	0.914	Pgl_GLEAN_10004673	NA
chr2_6916853	C/T	0.06	0.98	0.893	Pgl_GLEAN_10004674	NA
chr2_6916881	G/T	0.08	0.99	0.884		
chr2_6916927	A/G	0.09	0.98	0.870		
chr2_6916950	C/T	0.08	0.98	0.870		
chr2_6908254	G/A	0.97	0.03	0.926	Pgl_GLEAN_10004675	Far1-related sequence 12
chr2_6908787	A/C	0.07	0.98	0.901		
chr2_18356501	G/T	0.25	1.00	0.771	Pgl_GLEAN_10005795	NA
chr2_8847231	G/T	0.87	0.01	0.872	Pgl_GLEAN_10013745	NA
chr2_11155563	T/C	0.95	0.01	0.936	Pgl_GLEAN_10016106	Phytochrome C
chr2_236500005	G/A	0.98	0.05	0.923	Pgl_GLEAN_10020525	HAC1/HAC703
chr2_7820474	A/C	0.98	0.10	0.881	Pgl_GLEAN_10022823	NA
chr2_7823128	G/C	0.99	0.07	0.922	Pgl_GLEAN_10022824	NA
chr2_7823261	T/C	0.97	0.08	0.887		
chr2_4843975	C/A	1.00	0.06	0.944	Pgl_GLEAN_10033790	Heading date 16/Early flowering 1
chr5_63973729	A/C	0.09	0.97	0.880	Pgl_GLEAN_10011525	NA
chr5_63973809	G/T	0.11	0.99	0.899		

Frequencies (F) of the reference allele in LF and EF accessions are shown as well as observed *F*_ST_ values, gene name and associated annotation (NA indicates no annotation found).

### Genomic association analyses

**Figure 5:**
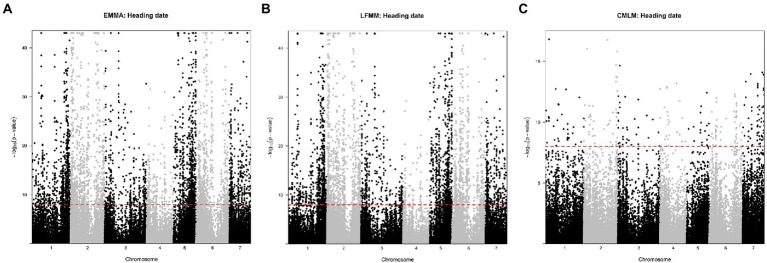
Manhattan plots of Fischer combined *p*-values for association with heading date. **(A)** with the EMMA approach, **(B)** with the LFMM approach, and **(C)** with the CMLM approach. Black and grey colors indicate chromosome. The red dashed line represents the 10^−8^ value considered for significance. Manhattan plots for the remaining traits are in the Supplementary Figure 5.

We applied a stringent threshold of 10^−8^ to declare that a SNP was significantly associated with a trait. All SNPs significant at this threshold were also significant when considering a FDR of 1%. The LFMM approach detected 5,017 SNPs out of the 96,881 SNPs tested. A total of 72% were associated with the heading date (Figure 5A). The EMMA approach detected 5,576 SNPs and 70% were associated with the heading date (Figure 5B). The CMLM approach detected 860 SNPs and 32% only were associated with the heading date while 46% were found associated with panicle length (Figure 5C). The Q-Q plots and value of p distributions suggested a good fit of all three models (Supplementary Figures 3, 4). Only the 315 SNPs detected by all three GWAS methods were further considered. Among those 315 SNPs, 24% were found on chromosome 2 and 16% on chromosomes 1 and 5. Most SNPs detected by all three methods were linked to length (67%) and diameter (13%) of the panicle. A total of 18 SNPs (6%) were found to be associated with the heading date and were found in 12 genes with some of them being annotated as part of the RAB GTPase family and two as cyclin-dependent protein serine/threonine kinase. Allele effect of these 18 SNPs on heading date was estimated (Supplementary Table 5). A median effect of +11.4 days for LFMM, +4.7 days for EMMA and + 1.9 days for CLMM was found. Despite these differences in values, rank of SNPs was conserved across methods. Two SNPs stand out with an estimated effect of +23 days with CMLM. Those SNPs are found in the gene *Pgl_GLEAN_10008450* on chromosome 2. This gene is encoding as a calcium dependent protein kinase (CDPK). Looking at the genotypes distribution of these SNPs, we observed that the LF accessions are found at the heterozygous state only while the EF are fixed for the alternate allele (Supplementary Figure 6). For diagnostic in breeding, lines with those SNPs (chr2_21407739, chr2_21407791 and chr2_7716171) can be used as donors/sources with, respectively, G/C, G/A, and TC favorable alleles for EF and LF group.

## Discussion

In Senegal, late-flowering landraces compared to early-flowering landraces are preferred for fodder as well as for roof and fence construction because of longer and more robust stems. This implied a genetic structuring and different selection pressure on these two types. Data reported here supported a clear genetic differentiation between EF and LF landraces. Meanwhile, a large fraction of genetic diversity (95%) is still segregating inside each flowering group. Genetic clustering was consistent with the flowering structure; other phenotypic findings were also significant. Short cycle landraces had longer and heavier panicles compared to long cycle landraces, which had longer stems and shorter panicles. This finding was consistent with those of previous studies (Marchais, 1982; Dancette, 1983; Zongo et al., 1988; Akanvou et al., 2012; Diack et al., 2020) and was in accordance with the farmer’s uses.

To identify key genes related to flowering time and relevant agronomic traits, two complementary approaches were used: (1) genome-wide selection scanning and (2) genome-wide association studies (GWAS). Conservative thresholds (PCAdapt: FDR of 0.001, BayeScan: prior odds of the model with selection to 10,000) were applied for both genome-scanning methods to pick up the strongest selection signature and identify key variants. GWAS models were used while taking the genetic structure into account. As in our case the genetic structure very closely matched the classification into EF and LF landraces, GWAS was expected to be more effective for detecting SNPs segregating within the flowering groups. For instance, SNPs detected by the genomic approaches were fixed in both groups with allelic frequencies >0.99 (Table 2), and thus could not be detected by the GWAS approaches, notably by the conservative CMLM method which seems to overcorrect the population structure. GWAS analysis was thus able to highlight other phenotypic traits and notably to detect a number of polymorphisms and genes associated with yield (Supplementary Tables 5, 6). Noteworthy, our combination of approaches appears to have been effective and relevant since we were able to detect genes that had been identified in previous studies with different samples and spatial scales [five genes identified by Diack et al. (2020) and Rhoné et al. (2020)], suggesting false discoveries were reduced and that our results could be mainstreamed to other pearl millet landraces.

In relation to flowering time, we highlighted fives relevant genes all found on chromosome 2: *PhyC* (*PHYTOCRHOME C*), *FRS12* (*FAR1-RELATED SEQUENCE 12*), *HEADING DATE 16* (*Hd16*)/*EARLY FLOWERING 1* (*EF1*), *HEADING DATE 3* (*Hd3a*) and *OsHAC1/OsHAC703* genes. The *PhyC* gene plays an important role in flowering time induction *via* photoperiodic cues in several cereal species (Izawa et al., 2002; rice: Takano et al., 2005; Ishikawa et al., 2011; wheat: Chen et al., 2014). *PhyC* has already been found to be a major flowering time gene in pearl millet (Saïdou et al., 2009, 2014; Vigouroux et al., 2011; Diack et al., 2017). In our study, SNP chr2_11155563 with is 4,745 bp away from the polymorphisms found in those previous studies, was detected by genomic scan approaches and in association with stem length by LFMM and EMMA methods. This gene was also reported to be associated with spike length and stem diameter in an inbred line panel (Saïdou et al., 2009), and with other traits including stay green, panicle diameter, panicle harvest index and panicle length under both well-watered and drought stress conditions (Sehgal et al., 2015; Debieu et al., 2018). Sequencing the entire *PhyC* gene from West African inbred lines would be interesting to investigate haplotypic diversity and the effect of the different variants of flowering time.

This is the first time that the role of *FRS12* in pearl millet flowering is highlighted. Loss-of-function of *FRS12* in *A. thaliana* results in early-flowering plants with overly elongated hypocotyls (Ritter et al., 2017). In pearl millet, the *FRS12* gene is located in a 530 kb region encompassing 12 genes detected in our study. This genomic region may represent an important quantitative trait locus (QTL) for flowering time. Although *FRS12* is a good candidate, we cannot overlook the possibility that other genes in this specific region could be directly causative of the phenotype. The gene *Pgl_GLEAN_10033790* is annotated as *HEADING DATE 16* (*Hd16*)/*EARLY FLOWERING 1* (*EF1*). The role of *Hd16* on flowering has been studied in rice (Dai and Xue, 2010) and linked to adaptation to high latitude (Hori et al., 2013). Interestingly, plants with non-functional *Hd16* have delayed flowering and heavy straw and panicle weight in paddy fields with low nitrogen input and no fertilizer application (Tanaka et al., 2019). [AA] pearl millet genotypes for the SNP chr2_4843975 showed a + 1.5 days delay in flowering and longer ([AA] 279 cm *vs* [CC] 240 cm) and thicker ([AA] 1.8 cm *vs* [CC] 1.6 cm) stems. Another strong candidate is the gene *Pgl_GLEAN_10020525,* which is annotated as *OsHAC1/OsHAC703* (histone acetyl transferase). This gene was also detected as associated to the heading date in pearl millet by Rhoné et al. (2020). By aligning on the reference genome the SSR markers used in the QTL analysis from (Kumar et al., 2017), we were able to find that the *HAC1* gene is part of QTL1:166 identified in that study as strongly involved in flowering. In *Arabidopsis HAC1* mutants flower late due to increased *FLOWERING LOCUS C (FLC*) expression (Deng et al., 2007). In rice, *OsHAC1* may be involved in the abscisic acid signaling pathway for responses to environmental stress during rice seedling growth, as well as to salt and drought stress (Liu et al., 2012; Fang et al., 2014). Kumar et al. (2017) study identified two additional QTLs on chromosome 3 linked to flowering, the QTL3/62 and the QTL3.100. The QTL3/62 encompasses the gene *Pgl_GLEAN_10027181* annotated as *CyclinT1;3* which was found to be associated with heading date in our study. The mean CMLM effect of the alternate allele of SNP chr3_295789863 is +1.53 days. The *Arabidopsis thaliana* homolog *AT1G27630* may be involved in pathway linking circadian clock and cell cycles. The QTL3/100 encompasses the gene *Pgl_GLEAN_10032681* that was detected in our PCAdapt analysis. This gene is annotated as an ethylene-responsive factor like protein (*ERFL1*) from the *APETALA2* family.

The GWAS analysis detected SNPs linked to yield through association with panicle and grain characteristics. The annotation of homologous genes in *Arabidopsis thaliana* revealed a number of potentially interesting genes which magnitude of effect seems to be also a function of the genetic background, i.e., early-vs. late-flowering landraces. Fourteen of these genes are annotated as being related to responses to abiotic stimuli (salt stress, water deprivation, osmotic stress) or related to the immune response. A total of 13 genes are related to reproduction. One of them is *Pgl_GLEAN_10001878* and it is annotated as *LORELEI* in *Arabidospis* thaliana (Tsukamoto et al., 2010). When looking at its effect, it seems that the presence of the alternate allele on SNP chr3_18486380, increases 1,000 seed weight (Supplementary Figure 6). A second gene was found to be associated with 1,000 seeds weight, *Pgl_GLEAN_10007024*. This gene annotated as a PGR5-like protein and it may be involved in photosynthesis (DalCorso et al., 2008) in *Arabidopsis*. A clear decrease of 1,000 seeds weight is noted when individuals carry the alternative allele on SNP chr7_55923381 (Supplementary Figure 6). We have detected four genes whose annotation was linked to root development. Three of them were detected by the GWAS approach through their association with panicle size. The gene *Pgl_GLEAN_10017314* is annotated as a global transcriptor factor in *Arabidopsis thaliana*. The alternate allele of SNP chr4_48223622 increases panicle diameter at the homozygous state (Supplementary Figure 6). Another of these genes is *Pgl_GLEAN_10003976*. This gene is homolog to the *ERULUS* gene in *Arabidopsis thaliana*. *ERULUS* gene has been described as a core root hair regulator, involved in the establishment of a functional apical [Ca^2+^] gradient (Bai et al., 2014; Kwon et al., 2018). The establishment of this gradient seems to impact the pollen tube growth and fertilization (Schoenaers et al., 2017).

## Conclusion

Two complementary genome-wide approaches, i.e., selection scanning and association analysis (GWAS) were used in this study with the main objective to identify key genes linked to flowering and agromorphologic traits in pearl millet, a major staple cereal in sub-Saharan Africa. To date, PhyC was the only clearly identified flowering-related gene. We were able to identify several other genes that appear to be as much as important, such as *FSR12* and *HAC1*. *HAC1* and two other genes appear to be part of QTLs identified in previous studies and deserve further investigation. At the same time, we were able to identify a large number of genes and variants that could contribute to the improvement of pearl millet yield, especially since their impact was demonstrated across flowering cycles.

## Data availability statement

The original contributions presented in the study are included in the article/supplementary material, further inquiries can be directed to the corresponding author/s.

## Author contributions

AB, CB-S, ADF, NK, and YV designed the study. AB, CB, CB-S, PC, ADF, and BR performed the analyses. AD, MC, CM, and LZ generated genomic datasets. AB, CB-S, AC, ADF, ALF, KO, DM, MT, and YV managed the field studies and generated phenotypic datasets. ADF and CB-S wrote the paper with all authors’ contributions. All authors contributed to the article and approved the submitted version.

## Funding

This project was supported by Agropolis Fondation under reference ID 1403–057 through the Investissements d’avenir programme (Labex Agro: ANR-10-LABX-0001-01) in the framework of I-SITE MUSE (ANR-16-IDEX-0006), the CERAO project (ANR-13-AGRO-002), while the research leading to these results received funding from the UK National Environment Research Council (NERC)/Department for International Development (DFID) Future Climate For Africa Program, under the AMMA-2050 project (grant numbers NE/M020002/1; NE/M019934/1). YV was also funded under the CRP on Dryland Cereals.

## Conflict of interest

The authors declare that the research was conducted in the absence of any commercial or financial relationships that could be construed as a potential conflict of interest.

## Publisher’s note

All claims expressed in this article are solely those of the authors and do not necessarily represent those of their affiliated organizations, or those of the publisher, the editors and the reviewers. Any product that may be evaluated in this article, or claim that may be made by its manufacturer, is not guaranteed or endorsed by the publisher.

## Key message

Tuning flowering time plays a key role for adaptation to future climate change. We show that *PhyC*, *FRS12* and *HAC1* are key genes in the pearl millet flowering cycle.
