# Supplementary material for: Genomic footprints of selection in early-and late-flowering pearl millet landraces
Source: Front Plant Sci. 2022 Oct 12;13:880631. doi: 10.3389/fpls.2022.880631 (PMC9597309; doi:10.3389/fpls.2022.880631)
Supplement: Supplementary file 1 [file Data_Sheet_1.DOCX]

Supplementary Material

# Supplementary Tables

**Table S1. SNP annotation**

The 196,580 SNPs were annotated using SNPeff 4.3 (Cingolani et al., 2012) and the genome annotation files available at http://dx.doi.org/10.5524/100192 (Varshney et al., 2017). We used an upstream and downstream interval of 1,000 bp from the start and end positions of the gene.

**Table S2. Gene annotation**

The table lists the 20,126 genes where SNPs were found. Annotation was done using the genome annotation files available at http://dx.doi.org/10.5524/100192 (Varshney et al., 2017) using GO, InterPro, Kegg, Swiss-Prot and TrEMBL databases.

**Table S3. Correlation values between the 11 phenotypic traits**

Correlation values are below and p-values above the diagonal. Bold p-values are not significant at 0.05

**Table S4. SNP diversity estimates**

Genetic measures: observed (*H_Obs_*) and expected (*H_Exp_*) heterozygosity and F_IS_ inbreeding coefficients are estimated for each SNP for early- and late-flowering groups. Genetic differentiation *F_ST_* measures between EF and LF groups are provided.

**Table S5. Detected SNPs**

The 369 SNPs detected by at least one method (PCAdapt, BayeScan, or the three GWAS approaches). The table provides the traits with significant associations (i.e. detected by the three GWAS methods with p-values <10^-8^) for each SNP; if detected by PCAdapt; if detected by BayeScan; and the SNP annotation.

**Table S6. Detected genes**

The 317 genes detected are listed with the number of SNPs detected; maximum number of methods detecting related SNPs; number of SNPs found in association for each trait; gene annotations and their potential *Arabidopsis thaliana* homolog and its annotation*.*

## Supplementary Figures

**Figure S1. Eigenvalues of PCA on the 11 phenotypic traits**

Mean phenotypic measures across nine repetitions for 11 traits of 109 accessions are used to perform a centered and scaled PCA. PC1 and PC2 explained 41.5% and 28% of the inertia, respectively.

**Figure S2. BayeScan plot of detected SNPs.**

We applied the BayeScan approach to EF and LF accession groups (pooling accessions for the four villages) on SNPs with MAF ≥ 0.05. We set the prior odds of the model with selection at 10,000, with a thinning interval of 20 and an FDR of 0.05, while BayeScan analysis identified 23 outlier SNPs, all of which were also detected in the PCAdapt analysis.

**Figure S3. Histograms of GWAS p-values**

For each trait and GWAS method (EMMA, LFMM, CMLM), histogram p-values are presented for the 9 replicates and for the Fischer’s combined probability test.

**Figure S4. QQplots**

For each trait and each GWAS method (EMMA, LFMM, CMLM), QQplots of p-values are presented for the 9 replicates and for the Fischer’s combined probability test.

**Figure S5.** Manhattan plots of Fischer’s combined p-values for association. For each trait and each GWAS method (EMMA, LFMM, CMLM), Manhattan plots are presented.

The red dashed line represents the 10^-8^ value considered for significance.

**Figure S6. Boxplots of phenotypic traits for each SNP genotype for early and late-flowering pearl millet accessions.**  Boxplots are presented for four SNPs: SNP Chr2_21407739, SNP Chr3_18486380, SNP Chr7_55923381, SNP Chr4_48223622. Genotypes are indicated as 0 for homozygote for the reference allele, 1 for heterozygotes and 2 for homozygotes of the alternate allele.
