# Supplementary figures and images for: Genomic footprints of selection in early-and late-flowering pearl millet landraces
Source: Front Plant Sci. 2022 Oct 12;13:880631. doi: 10.3389/fpls.2022.880631 (PMC9597309; doi:10.3389/fpls.2022.880631)

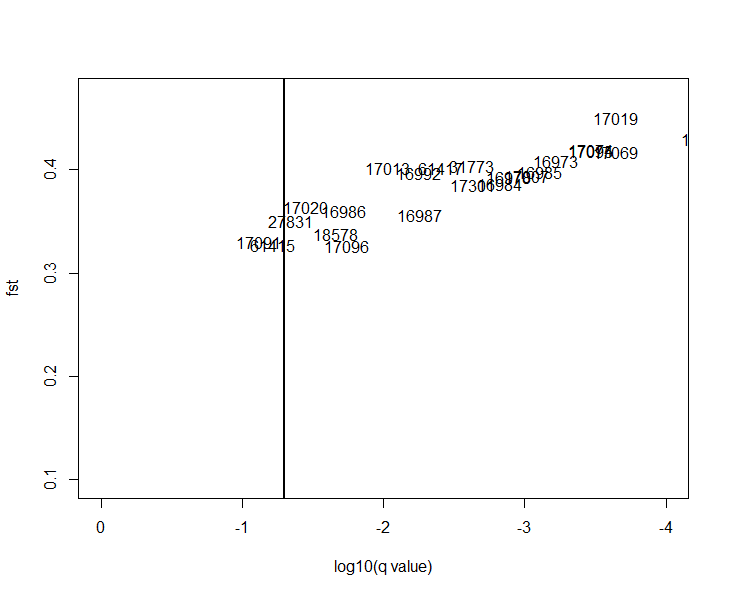

Supplement: Supplementary file 2 [file Image_1.PNG]

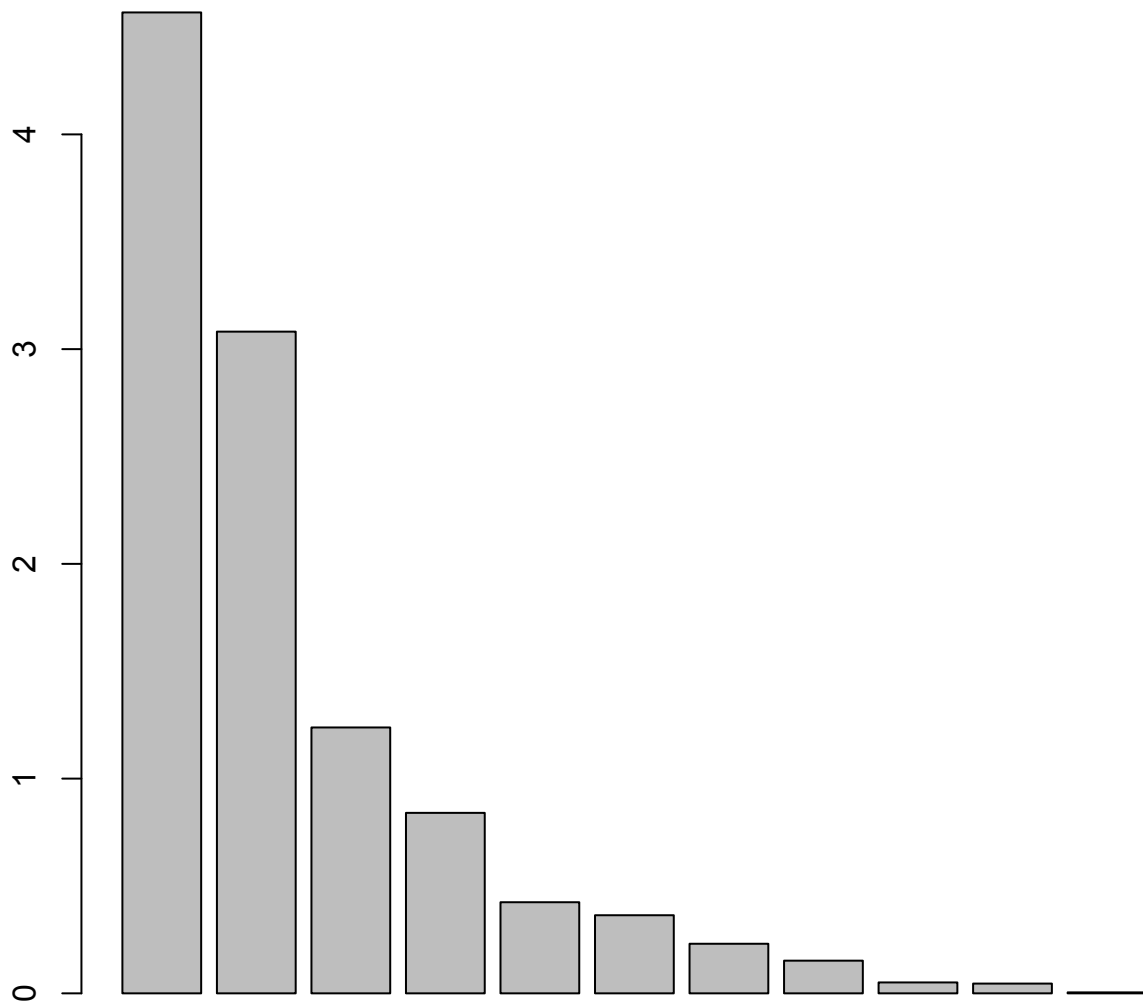

Supplement: Supplementary file 3 [file Image_2.PDF]
